# Lactic Acid Bacteria Ameliorate Diesel Exhaust Particulate Matter-Exacerbated Allergic Inflammation in a Murine Model of Asthma

**DOI:** 10.3390/life10110260

**Published:** 2020-10-28

**Authors:** Sun Woo Jin, Gi Ho Lee, Min Jung Jang, Gyeong Eun Hong, Jae Young Kim, Gi Deok Park, Hui Jin, Hyun Su Kim, Chul Yung Choi, Jae Ho Choi, Su Gwon Lee, Hye Gwang Jeong, Yong Pil Hwang

**Affiliations:** 1 Department of Toxicology, College of Pharmacy, Chungnam National University, Daejeon 34134, Korea; mpassword@cnu.ac.kr (S.W.J.); ghk1900@cnu.ac.kr (G.H.L.); 2 Department of Research, GREEN CROSS Wellbeing Co., Ltd., Seongnam 13595, Korea; mjjang@gccorp.com (M.J.J.); hge0121@gccorp.com (G.E.H.); jaeyoung4740@gccorp.com (J.Y.K.); pkd6300a@gccorp.com (G.D.P.); iuhnij@gccorp.com (H.J.); hs_kim4@gccorp.com (H.S.K.); 3 Jeonnam Bioindustry Foundation, Jeonnam Institute of Natural Resources Research, Naju 59338, Jeollanamdo, Korea; blockstar@hanmai.net; 4 Subtropical/tropical Organism Gene Bank, Jeju National University, Jeju 63243, Korea; chlkoala@naver.com; 5 Department of Pharmacokinetics, College of Pharmacy, Gachon University, Incheon 21936, Korea; club159@naver.com; 6 Department of Pharmaceutical Engineering, International University of Korea, Jinju 52833, Korea; 7 Fisheries Promotion Division, Mokpo City 58613, Jeollanamdo, Korea

**Keywords:** lactic acid bacteria, DEPM, allergic airway inflammation, anti-inflammation

## Abstract

Several air pollution components such as sulfur dioxide, ozone, nitrogen dioxide, and diesel exhaust particulate matter (DEPM) have been linked to the development of asthma. In this study, we investigated the therapeutic potential of three lactic acid bacteria species, *Lactobacillus plantarum* GREEN CROSS Wellbeing (GCWB)1001, *Pediococcus acidilactici* GCWB1085, and *Lactobacillus rhamnosus* GCWB1156, in preventing DEPM-exacerbated asthma in mice. BALB/c mice were first sensitized with ovalbumin (OVA) and were either challenged with OVA or DEPM (DEPM-exacerbated asthma model) by intranasal instillation. All three strains showed no hemolytic activity, suggesting a good safety profile. Oral administration of lactic acid bacteria reduced OVA + DEPM-induced inflammatory infiltration, goblet cell hyperplasia, airway remodeling, and the levels of proinflammatory cytokines and chemokines in bronchoalveolar lavage fluid (BALF). The probiotics also attenuated OVA + DEPM-induced immunoglobulin E (IgE) levels in serum and in BALF, and significantly reduced caspase-3 activity, total collagen level, and matrix metalloproteinase (MMP)-9 activity. In conclusion, lactic acid bacteria such as *L. plantarum* GCWB1001, *P. acidilactici* GCWB1085, and *L. rhamnosus* treatment in mice with asthma showed significant efficacy in preventing lung inflammation exacerbated by DEPM administration.

## 1. Introduction

The prevalence of respiratory diseases such as asthma and allergic rhinitis has increased in developed countries over the past 30 years. Bronchial asthma is a heterogeneous and chronic inflammatory lung disease characterized by reversible airway obstruction, airway hyperresponsiveness, and airway remodeling. It involves goblet cell hyperplasia, subepithelial fibrosis, and smooth muscle hypertrophy [1]. Asthma phenotypes vary by age, sex, and ethnicity and are influenced by a combination of environmental, epigenetic, and genetic factors [2,3,4]. Environmental factors such as allergens, air pollution, tobacco smoke, ozone, viruses, bacteria, and fungi can also enhance the risk of developing respiratory diseases.

Ambient air pollution caused by particulate matter (PM) poses a serious problem to human health worldwide. One of the major components of PM is diesel exhaust PM (DEPM). DEPM was designated as a group 1 human carcinogen in June 2012. It consists of gas, steam, and fine particles. The surfaces of these fine particles contain a large number of metals, such as zinc and copper, and polycyclic aromatic hydrocarbons, such as anthracene, naphthalene, and benzo(a)pyrene [5].

Recent studies have shown that intravenous or intratracheal administration of nano-sized particle-rich DEPM increases oxidative stress and inflammation. It can also cause acute and chronic airway diseases such as allergic rhinitis, bronchial asthma-like diseases, and lung cancer [6,7,8]. In addition, recent epidemiological studies have shown that fine PM (PM2.5) in ambient air is associated with an increased incidence of asthma [6,7,8].

In inflammatory diseases such as asthma, DEPM increases chemokine production [6,7,8]. An increase in immunoglobulin E (IgE) and T helper (Th) 2-type cytokines such as interleukin (IL)-4, IL-6, IL-10, and IL-13 have been reported [6]. These allergic mediators trigger cascades that can cause any of the following manifestations: airway inflammation, mucus production, activation of neutrophils, eosinophils, and macrophages, and bronchial smooth muscle contractions [7,8].

Probiotics have anti-inflammatory effects and anti-cancer effects, induce beneficial bacterial proliferation, suppress harmful bacteria, improve intestinal health, reduce blood cholesterol levels, and suppress endogenous infections [9,10,11,12]. Lactic acid bacteria are a probiotic that prevents the early development of allergic diseases in children and in mouse models of asthma [13,14]. These probiotics are beneficial because they promote T regulatory cell development and rebalance the immune response toward a Th1-dominant state [15]. In many studies, the oral administration of probiotics such as *Lactobacillus rhamnosus* GG, *Lactobacillus gasseri*, *Lactobacillus fermentum* NWS29, *Lactobacillus casei* NWP08, *Lactobacillus rhamnosus* NWP13, and *Lactobacillus salivarius* PM-A0006 have shown significant benefits in mouse models of allergic asthma [13,16,17,18]. In addition, a recent study demonstrated that *Bifidobacterium lactis* inhibits PM2.5-induced lung inflammation in mice [19]. Additionally, *L. rhamnosus* and *B. breve* can reduce the activation of human macrophages caused by cigarette smoke [20].

Recently, epidemiological studies revealed that PM, including DEPM, exacerbated asthma [21,22]. Therefore, it is important to determine effective and preventative strategies to avoid exacerbations in individuals with asthma who are constantly exposed to serious air pollution. Previous studies have evaluated the efficacy of probiotics in models of classic ovalbumin (OVA)-induced asthma [17,23,24]. However, it is still unclear whether probiotics are beneficial in managing DEPM exacerbations in animal models of asthma. In a preliminary study examining the anti-inflammatory effects of probiotics, we found three probiotics (*Lactobacillus plantarum* GREEN CROSS Wellbeing (GCWB)1001, *Pediococcus acidilactici* GCWB1085, and *Lactobacillus rhamnosus* GCWB1156, isolated from Korean kimchi) that exert a suppressive effect on tumor necrosis factor (TNF)-α and nitric oxide production in vitro. Therefore, in this study, we assessed the anti-inflammatory and anti-asthmatic effects of *L. plantarum* GCWB1001, *P. acidilactici* GCWB1085, and *L. rhamnosus* GCWB1156, isolated from Korean kimchi (fermented napa cabbage), in allergic responses induced by DEPM.

## 2. Results

### 2.1. L. plantarum GCWB1001, P. acidilactici GCWB1085, and L. rhamnosus GCWB1156 are Safe Probiotic Strains

To evaluate whether *L. plantarum* GCWB1001, *P. acidilactici* GCWB1085, and *L. rhamnosus* GCWB1156 are safe for probiotic use, we performed a hemolysis test using *E. coli* American Type Culture Collection (ATCC)25922, *Staphylococcus aureus* ATCC12600, and *Enterococcus faecalis* ATCC19433 as controls. *L. plantarum* GCWB1001, *P. acidilactici* GCWB1085, *L. rhamnosus* GCWB1156, and *E. faecalis* ATCC19433 did not have gamma hemolysis activity (Table 1). In contrast, *E. coli* ATCC25922 and *S. aureus* ATCC12600 showed alpha and beta hemolysis activities. The results demonstrated that *L. plantarum* GCWB1001, *P. acidilactici* GCWB1085, and *L. rhamnosus* GCWB1156 are safe to use as probiotics.

### 2.2. L. plantarum GCWB1001, P. acidilactici GCWB1085, and L. rhamnosus GCWB1156 Decrease the Weight of the Spleen and Lungs in a DEPM-Exacerbated Mouse Model of Asthma

**Figure 1:**
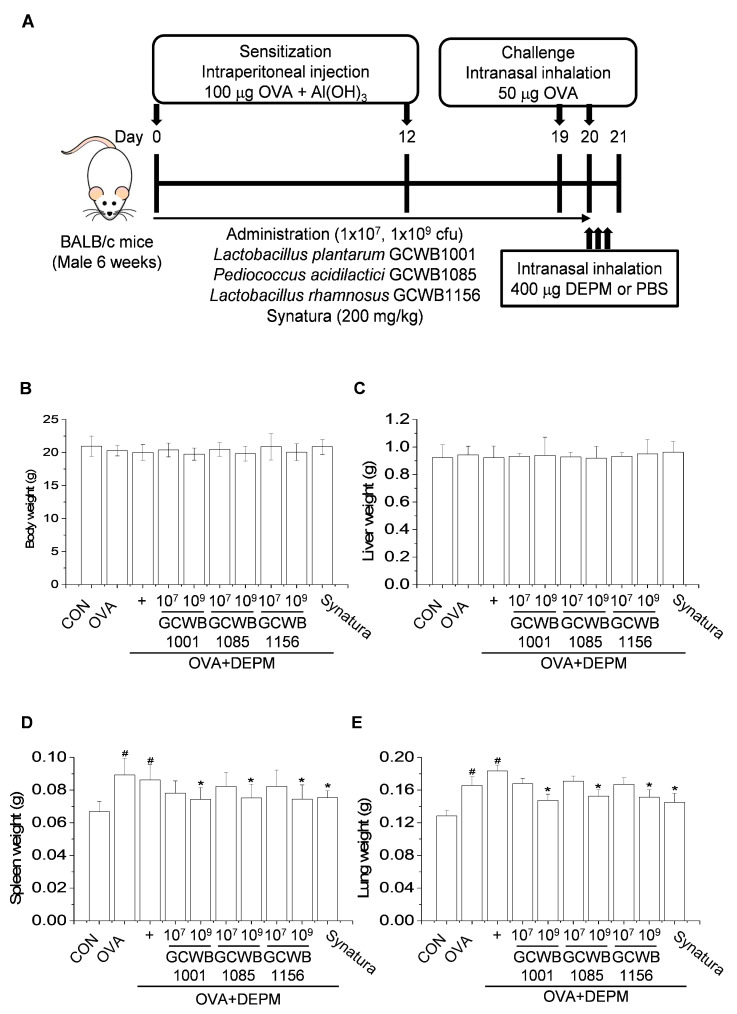
The effects of *L. plantarum* GCWB1001, *P. acidilactici* GCWB1085, and *L. rhamnosus* GCWB1156 on organ weight in a mouse model of asthma exacerbated by diesel exhaust particulate matter (DEPM). (**A**) A DEPM-exacerbated mouse model of asthma and lactic acid bacteria treatment was established. Mice were given probiotics daily from days 0 to 20. Mice were sensitized with ovalbumin (OVA) on days 0 and 12. Nasal challenge with OVA was initiated on days 19 and 20. After the last OVA challenge on day 20, mice were given an extra intranasal administration of DEPM solution three times over a 3-h interval. Mice were sacrificed on day 21, and tissue was collected for experimental analysis. (**B**–**D**) The effects of *L. plantarum* GCWB1001, *P. acidilactici* GCWB1085, and *L. rhamnosus* GCWB1156 on the weights of the body (**B**), liver (**C**), spleen (**D**), and lungs (**E**) of mice challenged with OVA plus DEPM. Organs were collected and washed with phosphate buffered saline. The OVA-induced organ weight was measured using a portable balance (Voyager^®^ Pro, OHAUS). Synatura was used as a positive control. Data are expressed as means ± standard deviation (SD; n = 8). # *p* < 0.001 compared with the control group. * *p* < 0.001 compared with the OVA plus DEPM group. CON: control; cfu: colony forming units.

The DEPM-exacerbated mouse model of asthma is summarized in Figure 1A. DEPM or OVA treatment did not have a significant effect on body or liver weight in mice (Figure 1B,C). The weight of the spleen and the lungs were slightly increased in mice that received OVA compared with the control group. *L. plantarum* GCWB1001, *P. acidilactici* GCWB1085, and *L. rhamnosus* GCWB1156 administration reduced the weights of the spleen and lungs (Figure 1D,E). The increases in total spleen and lung weight were inhibited by Synatura, a positive control, compared with mice that received OVA alone or OVA plus DEPM.

### 2.3. Administration of L. plantarum GCWB1001, P. acidilactici GCWB1085, and L. rhamnosus GCWB1156 Alleviated the Inflammatory Cell Infiltration Exacerbated by DEPM Exposure

**Figure 2:**
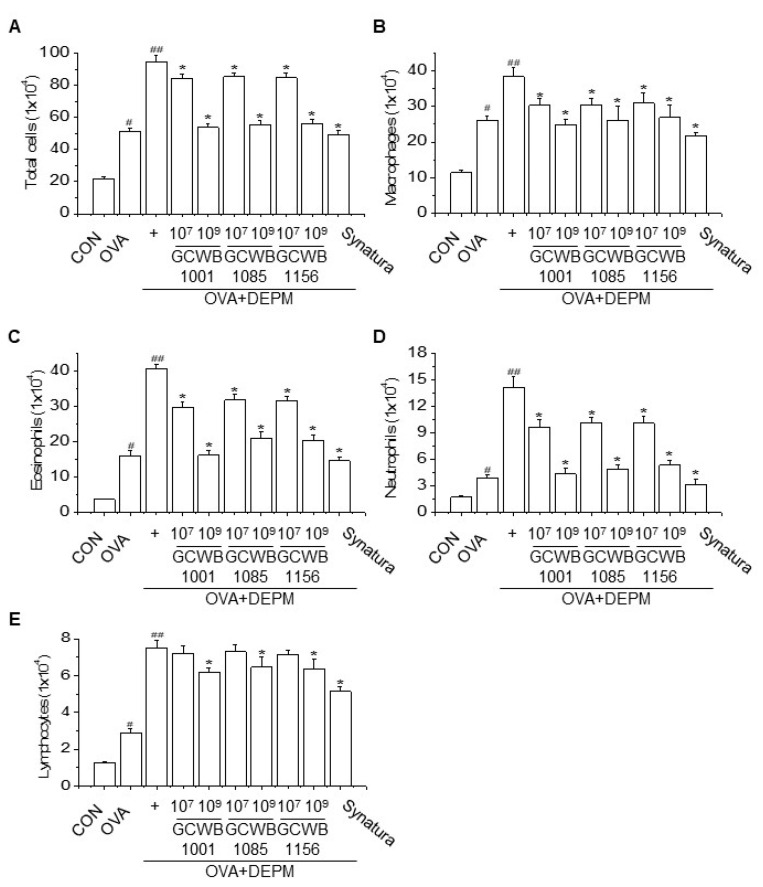
The effects of *L. plantarum* GCWB1001, *P. acidilactici* GCWB1085, and *L. rhamnosus* GCWB1156 on the cellular profile of bronchoalveolar lavage fluid (BALF) (**A**–**E**). Cell counts in BALF were obtained from all groups. The total cells and inflammatory cells (macrophages, neutrophils, eosinophils, and lymphocytes) were counted (× 10^4^) in millimeters by morphometric evaluations of cytospin preparations. At least 200 cells were examined in each cytospin. Synatura was used as a positive control. Data are expressed as means ± SD (n = 8). # *p* < 0.001 compared with the control group. ## *p* < 0.001 compared with the OVA group. * *p* < 0.001 compared with the OVA plus DEPM group.

Since inflammatory cell infiltration into the airways is a characteristic of allergic airway disorders, we evaluated the effects of *L. plantarum* GCWB1001, *P. acidilactici* GCWB1085, and *L. rhamnosus* GCWB1156 on inflammatory cell infiltration in bronchoalveolar lavage fluid (BALF). The cellular profile of BALF was investigated 24 h after the last intranasal injection (Figure 2). The combination of OVA plus DEPM stimulation significantly increased the number of macrophages, neutrophils, eosinophils, and lymphocytes compared with the control group and the OVA only group. Treatment with *L. plantarum* GCWB1001, *P. acidilactici* GCWB1085, and *L. rhamnosus* GCWB1156 also significantly decreased the numbers of macrophages, neutrophils, eosinophils, and lymphocytes in the BALF compared with mice stimulated with OVA plus DEPM (Figure 2). Synatura as a positive control also inhibited inflammatory cell infiltration compared with the OVA only group and the OVA plus DEPM group. These results suggest that *L. plantarum* GCWB1001, *P. acidilactici* GCWB1085, and *L. rhamnosus* GCWB1156 inhibit lung inflammation in an OVA-induced DEPM-exacerbated mouse model of asthma.

### 2.4. Administration of L. plantarum GCWB1001, P. acidilactici GCWB1085, and L. rhamnosus GCWB1156 Inhibited DEPM-induced Exacerbation of Inflammatory Cytokines and Chemokines in BALF

To investigate the effects of *L. plantarum* GCWB1001, *P. acidilactici* GCWB1085, and *L. rhamnosus* GCWB1156 on lung inflammation induced by OVA and exacerbated by DEPM, the levels of proinflammatory cytokines and chemokines in BALF were measured 24 h after the final instillation. As shown in Table 2, the levels of IL-4 and IL-13 (Th2-related cytokines) were significantly increased, whereas the level of interferon gamma (IFN-γ, a Th1-related cytokine) was decreased in the BALF of mice that received OVA compared with the control group. Furthermore, mice stimulated with OVA plus DEPM had significantly higher levels of proinflammatory cytokines (TNF-α, IL-1β, and IL-6) and chemokines (including monocyte chemoattractant protein-1 (MCP-1)) compared with both the control group and the OVA only group. A higher dose of *L. plantarum* GCWB1001, *P. acidilactici* GCWB1085, and *L. rhamnosus* GCWB1156 decreased the levels of TNF-α, IL-1β, IL-4, IL-6, IL-13, and MCP-1, but increased the levels of IFN-γ in BALF (Table 2). Similar effects were observed with Synatura.

### 2.5. Administration of L. plantarum GCWB1001, P. acidilactici GCWB1085, and L. rhamnosus GCWB1156 Inhibited the IgE Level Increased by OVA Plus DEPM in BALF and Serum

**Figure 3:**
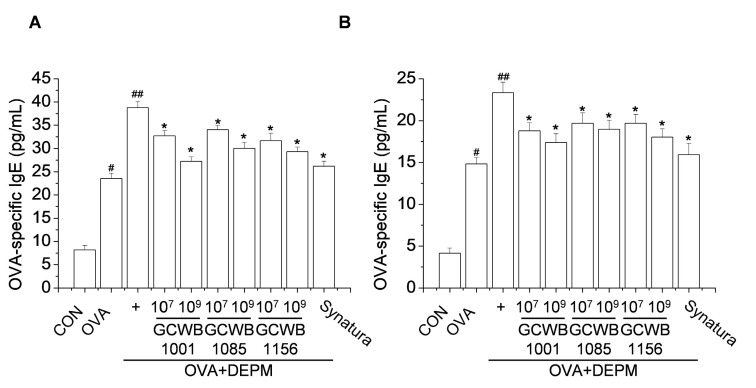
The effects of *L. plantarum* GCWB1001, *P. acidilactici* GCWB1085, and *L. rhamnosus* GCWB1156 on ovalbumin (OVA)-specific immunoglobulin E (IgE) levels in bronchoalveolar lavage fluid (BALF) and serum. The OVA-specific IgE levels in BALF (**A**) and serum (**B**) were analyzed by ELISA. Synatura was used as a positive control. Data are expressed as means ± SD (*n* = 8). # *p* < 0.001 compared with the control group. ## *p* < 0.001 compared with the OVA group. * *p* < 0.001 compared with the OVA plus diesel exhaust particulate matter (DEPM) group.

To investigate the effects of *L. plantarum* GCWB1001, *P. acidilactici* GCWB1085, and *L. rhamnosus* GCWB1156 on lung inflammation induced by OVA and exacerbated by DEPM, OVA-specific IgE levels in BALF and serum were measured by enzyme-linked immunosorbent assay (ELISA). As shown in Figure 3, the levels of OVA-specific IgE in BALF and serum were increased in the OVA-treated group compared with the control group. In contrast, treatment with *L. plantarum* GCWB1001, *P. acidilactici* GCWB1085, and *L. rhamnosus* GCWB1156 decreased OVA-specific IgE levels in BALF and serum compared with the untreated OVA-induced group in a dose-dependent manner. We observed similar effects with Synatura.

### 2.6. The Effects of L. plantarum GCWB1001, P. acidilactici GCWB1085, and L. rhamnosus GCWB1156 on the Inflammatory Response and Mucus Production in Lungs Caused by DEPM

**Figure 4:**
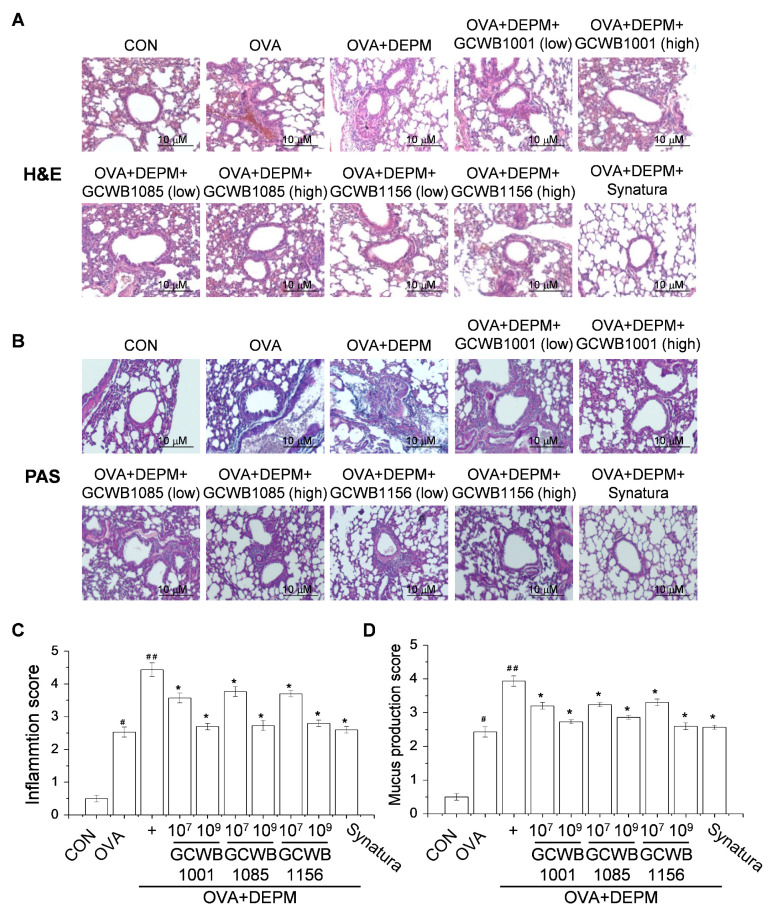
The effects of *L. plantarum* GCWB1001, *P. acidilactici* GCWB1085, and *L. rhamnosus* GCWB1156 on histopathological changes in a diesel exhaust particulate matter (DEPM)-exacerbated mouse model of asthma. Lung tissues were collected and fixed in 10% formaldehyde. Thin sections (4 um) were cut and stained with (**A**) hematoxylin and eosin (H&E) or (**B**) alcian blue/periodic acid–Schiff (PAS). Inflammatory cell infiltration (**C**) and mucus production (**D**) in lung tissues were scored as described in the Materials and Methods section. Synatura was used as a positive control. Data are expressed as means ± SD (n = 8). # *p* < 0.001 compared with the control group. ## *p* < 0.001 compared with the OVA group. * *p* < 0.001 compared with the OVA plus DEPM group. Magnification: 200×.

To evaluate the effects of *L. plantarum* GCWB1001, *P. acidilactici* GCWB1085, and *L. rhamnosus* GCWB1156 on lung inflammation and mucus production induced by OVA and exacerbated by DEPM, lung pathology was examined in the mice (Figure 4). Asthmatic lungs of mice treated with OVA alone or OVA plus DEPM had a higher infiltration of inflammatory cells in peribronchiolar and perivascular lesions compared with the control group (Figure 4A,C). Oral administration of *L. plantarum* GCWB1001, *P. acidilactici* GCWB1085, and *L. rhamnosus* GCWB1156 decreased the level of inflammatory cell infiltration, as analyzed by hematoxylin and eosin (H&E) staining (Figure 4A,C). Figure 4B and D show the bronchus in the lungs stained with alcian blue/periodic acid–Schiff (PAS). No pathologic alterations were found in the lungs of the control mice. Lungs from mice that received OVA or OVA plus DEPM exhibited an overproduction of mucus with goblet cell hyperplasia, as measured by PAS staining (Figure 4B,D). They also had higher goblet cell proliferation and mucus hypersecretion compared with control mice (Figure 4B,D). Oral administration of *L. plantarum* GCWB1001, *P. acidilactici* GCWB1085, and *L. rhamnosus* GCWB1156 inhibited goblet cell hyperplasia and mucus hypersecretion compared with untreated OVA-induced mice (Figure 4B,D). These pathological changes were slightly alleviated by Synatura treatment.

### 2.7. The Effects of L. plantarum GCWB1001, P. acidilactici GCWB1085, and L. rhamnosus GCWB1156 on Caspase-3 Activity, Total Collagen Level, and Matrix Metalloproteinase (MMP)-9 Activity in Lungs Treated with DEPM

**Figure 5:**
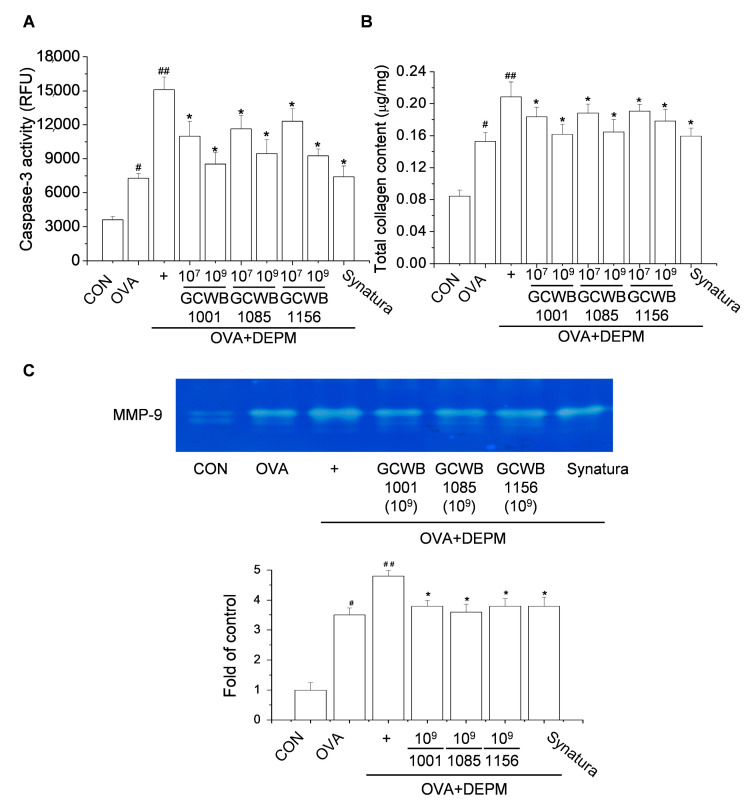
The effects of *L. plantarum* GCWB1001, *P. acidilactici* GCWB1085, and *L. rhamnosus* GCWB1156 on ovalbumin (OVA)-induced oxidative stress and lung tissue remodeling. (**A**) Caspase-3 activity was detected in tissue lysates using a fluorescence kit. (**B**) The level of lung collagen was measured using a Sircol collagen assay kit. (**C**) matrix metalloproteinase (MMP)-9 activity in bronchoalveolar lavage fluid (BALF) was analyzed by gelatin zymography. The results shown were obtained from three independent experiments. RFU: Relative fluorescence units. # *p* < 0.001 compared with the control group. ## *p* < 0.001 compared with the OVA group. * *p* < 0.001 compared with the OVA plus DEPM group.

The DEPM-induced increase in inflammatory activity has been correlated to the oxidative stress response [25]. We tested the effects of *L. plantarum* GCWB1001, *P. acidilactici* GCWB1085, and *L. rhamnosus* GCWB1156 on the activity of caspase-3 in the DEPM-exacerbated mouse model of asthma. Mice that received OVA or OVA plus DEPM showed higher caspase-3 activity compared with the control mice. Mice treated with *L. plantarum* GCWB1001, *P. acidilactici* GCWB1085, and *L. rhamnosus* GCWB1156 showed significantly lower caspase-3 activity compared with untreated mice (Figure 5A).

In chronic asthma, various cytokines promote airway epithelial cell proliferation and secrete polymorphic factors. These factors act synergistically on smooth muscle cells and subcutaneous fibroblasts to induce airway epithelial fibrosis, airway remodeling, and underground membrane thickness. Therefore, we assessed the total lung collagen level in the DEPM-exacerbated mouse model of asthma.

Mice treated with OVA alone or OVA plus DEPM had a higher total lung collagen level (Figure 5B). Following treatment with *L. plantarum* GCWB1001, *P. acidilactici* GCWB1085, and *L. rhamnosus* GCWB1156, the level of peribronchial fibrosis was significantly reduced. Numerous studies have shown that MMP-9 is involved in asthma and is associated with the gradual remodeling of the airway walls [26,27]. In this study, MMP-9 expression was significantly higher in the lung tissues of mice challenged with OVA (Figure 5C). This exacerbation was greater in mice exposed to both DEPM and OVA compared with OVA only. Mice treated with *L. plantarum* GCWB1001, *P. acidilactici* GCWB1085, *L. rhamnosus* GCWB1156, or Synatura had significantly lower MMP-9 activity compared with untreated mice stimulated with OVA plus DEPM (Figure 5C). These results suggest that *L. plantarum* GCWB1001, *P. acidilactici* GCWB1085, and *L. rhamnosus* GCWB1156 help restore the architecture of airway and lung tissue in asthmatic mice.

## 3. Discussion

The usefulness of naturally derived anti-asthmatic agents to treat patients with severe allergic asthma have recently gained attention [28,29]. Therefore, probiotics have been suggested as new agents for asthma therapy or as alternative medicine. In this study, we evaluated the effects of three orally administered lactic acid bacterial strains in a mouse model of asthma. More specifically, *L. plantarum* GCWB1001, *P. acidilactici* GCWB1085, and *L. rhamnosus* GCWB1156 alleviated the elevated airway inflammation and airway remodeling attributed to DEPM exposure in an OVA-induced mouse model of asthma.

The use of probiotics as a therapy requires careful consideration with regard to hemolytic activity and the potential to transfer antibiotic resistance genes. Probiotic microorganisms should not cause hemolysis or gelatin liquefaction in the host. In this study, *L. plantarum* GCWB1001, *P. acidilactici* GCWB1085, and *L. rhamnosus* GCWB1156 (all non-pathogenic Gram-positive bacteria) did not exert a hemolytic effect on cells. Further studies are needed to determine whether antibiotic resistance genes can cause acquired resistance at the gene level using polymerase chain reaction or whole genome sequencing.

Inflammation is associated with the pathogenesis of various diseases. It is considered a pivotal factor in the development and progression of asthma. Asthma is typically characterized by an imbalance between Th1 and Th2 immune responses, with a predominant shift towards Th2 reactions [6,30]. Previous studies have shown that *L. rhamnosus* GG induces Th1-predominant reactions during allergen sensitization, maintains the balance between Th1 and Th2 immune responses, and inhibits the production of IgE and Th2 cytokines from allergens [19,31]. Liu et al. and Wang et al. evaluated the anti-allergic effects of *L. plantarum* K37 and *L. paracasei* L9 on allergic responses and airway hyperresponsiveness. They found that K37 and L9 effectively reduced allergic responses in BALB/c mice with OVA-induced asthma by shifting towards a Th1-predominant response [21,32]. During homeostatic periods, gut microbiota and T cells within the gut mucosa engaged in profitable crosstalk, which can shape the systemic immune response of the individual [33]. T cell receptors are reactive to microbiota-derived antigens that are necessary for the adequate maturation of the immune system and to ensure proper colonization of the gut lumen [33]. Previous studies have shown that *Lactobacillus reuteri* attenuated allergic inflammation induced by house dust mites in mice and modulated gut microbes [34]. This study showed that oral administration of *L. plantarum* GCWB1001, *P. acidilactici* GCWB1085, and *L. rhamnosus* GCWB1156 can suppress IgE levels in BALF or serum. The probiotics also reduced the levels of TNF-α, IL-1β, IL-6, and MCP-1. Moreover, the probiotics attenuated macrophage, eosinophil, lymphocyte, and neutrophil infiltration in the lungs.

Oxidative stress also causes structural changes in asthmatic airways [35,36]. In the progression of asthma, goblet cell hyperplasia increases epithelial damage via endogenous and exogenous reactive oxygen species (ROS) [37,38]. Oxidative stress also enhances hypertrophy and proliferation of smooth muscle cells in the pulmonary vasculature [39]. Oxidative stress can be stimulated by polycyclic aromatic hydrocarbons and metals, which play an important role in causing allergic airway disease [26,39,40]. Polycyclic aromatic hydrocarbons and metals are thought to be the most harmful compounds in DEPM. Cumulative evidence suggests that ROS-mediated oxidative stress caused by DEPM plays a central role in the pathogenesis of allergic asthma [25]. Powerful antioxidants not only prevent the development of structural changes in the airways, but also mitigate established airway remodeling. In this study, *L. plantarum* GCWB1001, *P. acidilactici* GCWB1085, and *L. rhamnosus* GCWB1156 decreased caspase-3 activity in the OVA-induced airway inflammatory reaction. Synatura was demonstrated to be efficacious in decreasing cough and sputum in patients with acute upper respiratory infection and chronic inflammatory bronchitis in a phase III clinical trial conducted in 2010 (National Clinical Trial (NCT)01151202). Synatura was approved by the Korean Ministry of Food and Drug Safety in March 2011 for treating cough and sputum due to bronchitis. In addition, Synatura improved the quality of life in patients with chronic bronchitis-type chronic obstructive pulmonary disease, and significantly reduced fibrinogen levels [41].

There is increasing information on the availability and functionality of probiotics. The beneficial effects of probiotics are achieved via various mechanisms. It was reported that heat-killed and live *L. rhamnosus* GG have anti-inflammatory effects, and that some bacterial components of heat-killed *L. rhamnosus* GG were also effective [42]. The antioxidant effects of these probiotics have also been reported. For example, *L. acidophilus* 606 and *Lactobacillus gasseri* SBT2055 exhibited anti-oxidative effects in vitro [43,44]. In addition, in mice fed a high-fat diet, the oral administration of *L. plantarum* FC225 increased superoxide dismutase and glutathione peroxidase activities, while decreasing the malondialdehyde level, which is an indicator of oxidative stress [45].

Airway wall remodeling is characterized by hyperplasia, goblet cell proliferation, and collagen deposition [26,27]. MMPs are expressed in the airway during periods of airway remodeling [27]. Our results showed that mice treated with *L. plantarum* GCWB1001, *P. acidilactici* GCWB1085, and *L. rhamnosus* GCWB1156 exhibited attenuated airway inflammation, mucus production, collagen levels, and MMP-9 activity. In recent studies, particulate matter has been shown to induce new cellular and molecular mediators such as Th17 cells and IL-17A in the lungs of exposed mice [46]. IL-17A has been discovered to play an important role in more severe asthma phenotypes [47]. Thus, it is critical to find effective preventive strategies for those diagnosed with pre-existing asthma who are also consistently exposed to serious air pollution. However, the mechanism has not yet been identified, and further studies should be conducted to explore the therapeutic effects and side effects.

In conclusion, *L. plantarum* GCWB1001, *P. acidilactici* GCWB1085, and *L. rhamnosus* treatment in mice with asthma showed significant efficacy in preventing lung inflammation exacerbated by DEPM administration.

## 4. Materials and Methods

### 4.1. Chemicals and Reagents

ELISA kits for TNF-α, IL-1β, IL-4, IL-6, IL-13, IFN-γ, and MCP-1 were purchased from R&D Systems (Minneapolis, MN, USA). The Sircol Collagen Assay kit was purchased from Biocolor Ltd., (Belfast, Northern Ireland). The Caspase-3 Activity Assay Kit was obtained from Cell Signaling Technology (Beverly, MA, USA). The OVA-specific-IgE ELISA kit and diesel particulate matter NIST^®^ SRM^®^ 2975 were obtained from Sigma-Aldrich (St. Louis, MO, USA). All kits were used according to the manufacturers’ protocols. All chemicals were of the highest commercially available grade.

### 4.2. Preparation of DEPM Suspensions

The National Institute of Standard and Technology standard reference material (SRM 2975) was purchased from Sigma-Aldrich. SRM 2975 is DEPM collected from a diesel-powered industrial forklift. A detailed description of the particles can be viewed at the National Institute of Standard and Technology. The DEPM stock solution (1 mg/mL) was suspended in sterile phosphate-buffered saline (PBS; Life Technologies, Carlsbad, CA, USA) and sonicated for 2 min in an ice bath using a sonicator (Sibata; OGAWA SEIKI, Tokyo, Japan) to minimize particulate aggregation.

### 4.3. Preparation of Lactic Acid Bacteria

*Lactobacillus plantarum* GCWB1001, *Pediococcus acidilactici* GCWB1085, and *Lactobacillus rhamnosus* GCWB1156 were isolated from Korean kimchi (fermented napa cabbage) and filed by GREEN CROSS Wellbeing (GCWB) (Seongnam, Korea). *Lactobacillus plantarum* GCWB1001, *Pediococcus acidilactici* GCWB1085, and *Lactobacillus rhamnosus* GCWB1156 were deposited in the Korea Culture Center of Microorganisms (KCCM) under registration numbers KCCM12698P (GCWB1001), KCCM12699P (GCWB1085), and KCCM12700P (GCWB1156). The strains were cultured in De Man, Rogosa, and Sharpe (MRS) broth (Sigma-Aldrich) under anaerobic conditions at 37 °C for 24 h. The bacterial cells in the stationary phase were centrifuged at 8000× *g* for 20 min at 4 °C. The cell pellet was washed twice with sterile PBS and adjusted to the appropriate density for in vivo experiments.

### 4.4. Measurement of Cell Viability

The quantity of live GCWB1001, GCWB1085, and GCWB1156 (1 × 10^11^ CFU/g freeze-dried lactobacilli) were quantified using 10-fold serial dilutions and the plate-count method established by the Ministry of Food and Drug Safety. Cell density during culturing in either the batch culture was analyzed by real-time measurements of optical density at 660 nm (G10S UV-Vis; Scinco Co., Gangnam, Korea).

### 4.5. Establishment of a DEPM-Exacerbated Mouse Model of Asthma and Probiotic Treatment

Mice were acclimatized for at least 1 week prior to the experiments. Six-week-old male BALB/c mice (20 ± 2 g) were obtained from Samtako (Osan, Korea) and housed in a room with controlled temperature (22 ± 2 °C) and humidity (50 ± 5%) under a 12 h light/dark cycle with free access to food and water. The mice used in this study were handled in accordance with the Guidelines for the Care and Use of Laboratory Animals published by the U.S. National Institutes of Health (NIH publication no. 85–23, 1996). All experimental procedures were approved by the Committee on Ethics of Animal Experiments of the International University of Korea (ethics no. IUK-2019-10-2). The mouse model was based on a classic 21-day OVA-sensitized and challenged mouse model of asthma, followed by the intranasal administration of the DEPM-extracted solution [19] (Figure 1A). Seven days after acclimatization, mice were divided into three groups (n = 8 per group): PBS-sensitized and challenged control group, OVA-sensitized and challenged group (OVA group), and OVA plus DEPM group. All groups except for the control group were intraperitoneally injected with 200 μL aluminum hydroxide (Al(OH)3) and saline (1:1) containing 100 μg OVA (Sigma-Aldrich, Grade V) on day 0 and 12. The control group received Al(OH)3 and saline. On days 19 and 20, the control group and other groups were challenged with either 50 μL saline or the same dose of OVA (50 µg/50 μL per mouse) by intranasal instillation. After the last OVA challenge on day 20, mice in the OVA plus DEPM group were given an extra intranasal administration of 50 μL DEPM solution (400 μg/50 μL per mouse) three times over a 3 h interval. The control and OVA group were given an extra intranasal administration of 50 μL PBS three times over a 3 h interval. All mice were anesthetized with urethane for intraperitoneal injections. Based on the OVA-induced DEPM-exacerbated mouse model of asthma, mice were fed either *L. plantarum* GCWB1001 (1 × 10^7^, 1 × 10^9^ cfu/head), *P. acidilactici* GCWB1085 (1 × 10^7^, 1 × 10^9^ cfu/head), *L. rhamnosus* GCWB1156 (1 × 10^7^, 1 × 10^9^ cfu/head), Synatura (200 mg/kg), or the same volume of PBS by gavage from the day of first sensitization (day 0) until the endpoint, over 20 days during sensitization (Figure 1). Synatura was used as a positive control. Synatura (AGNPP709) was developed by a South Korean pharmaceutical company (Ahn-Gook Pharmaceuticals Co., Ltd.), and formulated with a dried 30% ethanolic extract of *Hedera helix* L. (ivy; Lamiaceae) leaves and a dried water-saturated butanolic extract of the *Coptis chinensis* Franch (Ranunculaceae) rhizome (3:1, *w*/*w*) [48].

### 4.6. BALF Collection and Analysis of Cell Composition

BALF was collected immediately from mice following euthanasia by cervical dislocation, as described previously [49]. BALF was collected by flushing 1 mL PBS into the lung via the trachea immediately after sacrifice. Approximately 0.8 mL BALF was recovered after five lavages. BALF was placed on ice and centrifuged at 400× *g* for 5 min at 4 °C. The supernatants were collected to measure cytokine levels. The pellets suspended in PBS were used to assess cell composition. Total cell numbers were counted using a cytospin. Differential cell counts of macrophages, eosinophils, neutrophils, and lymphocytes were performed following staining with Giemsa solution (Sigma-Aldrich). Cells were analyzed according to general leukocyte morphology, and 200 cells were analyzed in each of four random locations.

### 4.7. Measurement of Hemolytic Activity

Lactic acid bacterial strains were grown in MRS broth culture overnight and streaked on blood agar plates (Sigma-Aldrich). They were then incubated at 37 °C for 72 h. Plates were observed for the formation of either clear zones (β-hemolysis), green hemolytic zones (α-hemolysis), or no zone (γ-hemolysis) around the *Lactobacillus* and *Pediococcus* colonies. A hemolysis test using the following bacteria with known hemolytic activity was performed as a control: *E. coli* ATCC25922, *S. aureus* ATCC12600, and *E. faecalis* ATCC19433.

### 4.8. Measurement of the Total Lung Collagen Level and Caspase-3 Activity

The total lung collagen level was determined using the Sircol Collagen Assay Kit (Biocolor Ltd., Belfast, Northern Ireland) according to the manufacturer’s protocols. The Caspase-3 Activity Assay Kit (Cell Signaling Technology, Beverly, MA, USA), a fluorescent assay that detects the activity of caspase-3 in tissue lysates, was used to measure caspase activity. The supernatants were collected and incubated with Ac-Asp-Glu-Val-Asp-7-Amino-4-methylcoumarin (Ac-DEVD-AMC) as a substrate at 37 °C. During the assay, activated caspase-3 cleaves this substrate between DEVD and AMC, generating highly fluorescent AMC, which is detected using a fluorescence reader at an excitation wavelength of 380 nm and an emission wavelength of 420–460 nm.

### 4.9. Histological Examination of Lung Tissue

Left lung lobes were excised and fixed in 4% paraformaldehyde for 24 h. The fixed lungs were dehydrated, paraffin-embedded, and sectioned at a thickness of 4 µm. The embedded tissue was then stained with H&E and alcian blue/PAS. Morphology and inflammation were analyzed under a light microscope (Zeiss Inc., Germany). Pulmonary histopathological changes were assessed using a five-point scoring system by three independent examiners. Sections were scored as follows: 0, no inflammatory cells; 1, minimal accumulation of inflammatory cells; 2, moderate accumulation of inflammatory cells; 3, severe accumulation of inflammatory cells; 4, extreme accumulation of inflammatory cells. Infiltrated goblet cells were assessed on a five-point scoring system by three independent examiners. Sections were scored as follows: 0, no goblet cells; 1, minimal infiltration of goblet cells; 2, moderate infiltration of goblet cells; 3, severe infiltration of goblet cells; 4, extreme infiltration of goblet cells [50].

### 4.10. ELISA

The concentrations of TNF-α, IL-1β, IL-4, IL-6, IL-13, IFN-γ, MCP-1, and IgE in BALF or serum were analyzed using DuoSet ELISA kits according to the manufacturer’s instructions. Each concentration was calculated using a linear regression equation based on the standard absorbance values. The optical density was measured at 450 nm using a Bio-Rad ELISA reader.

### 4.11. Enzymatic Activity of MMP-9 by Gelatin Zymography

The enzymatic activity of MMP-9 was analyzed by gelatin zymography. BALF from mice treated with lactic acid bacteria was electrophoresed on an 8% SDS–PAGE gel containing 0.2% gelatin. Gels were washed twice with washing buffer (50 mM Tris–HCl, pH 7.5, 100 mM NaCl, 2.5% Triton X-100), briefly rinsed in washing buffer without Triton X-100, and then incubated with incubation buffer (50 mM Tris–HCl, pH 7.5, 150 mM NaCl, 10 mM CaCl2, 0.02% NaN3) at 37 °C. After incubation, the gel was stained with 0.25% Coomassie Brilliant Blue G250 (Sigma-Aldrich) and then de-stained. MMP activity was represented by a clear zone of gelatin digestion.

### 4.12. Statistical Analysis

Results are expressed as means ± standard deviation (SD) of triplicate experiments. Data from the animal study are expressed as means ± SD (n = 8). Mean differences were evaluated by analysis of variance followed by Dunnett’s post hoc test, and *p* values < 0.05 were considered statistically significant.

## Figures and Tables

**Table 1:** Hemolytic activity of different probiotic strains.

	Strain	Positive		Negative
		Alpha	Beta	Gamma
Control	*Escherichia coli* ATCC25922	O		
	*Staphylococcus aureus* ATCC12600		O	
	*Enterococcus faecalis* ATCC19433			O
Test	*Lactobacillus plantarum* GCWB1001			O
	*Pediococcus acidilactici* GCWB1085			O
	*Lactobacillus rhamnosus* GCWB1156			O

ATCC: American Type Culture Collection; GCWB: GREEN CROSS Wellbeing.

**Table 2:** The effects of *L. plantarum* GCWB1001, *P. acidilactici* GCWB1085, and *L. rhamnosus* GCWB1156 on cytokine and chemokine levels in a diesel exhaust particulate matter (DEPM)-exacerbated mouse model of asthma.

	Group	TNF-α	IL-6	IL-1β	IL-4	IL-13	MCP-1	IFN-γ
OVA+ DEPM+	CON	68 ± 3.1	19 ± 0.4	283 ± 24	12 ± 0.2	282 ± 73	120 ± 14	149 ± 17
	OVA	562 ± 41 #	169 ± 26 #	610 ± 40 #	14.7 ± 0.8 #	2009 ± 307 #	745 ± 58 #	89 ± 8.1 #
	OVA+DEPM	1381 ± 312 ##	1028 ± 52 ##	1028 ± 52 ##	14.2 ± 0.6 #	4498 ± 994 ##	755 ± 105 ##	81 ± 8.8 #
	GCWB1001-low	884 ± 63 *	429 ± 88 *	889 ± 41 *	13.5 ± 0.4	2690 ± 438 *	507 ± 49 *	100 ± 7.3 *
	GCWB1001-high	743 ± 29 *	119 ± 19 *	795 ± 61 *	12.6 ± 0.3 *	1795 ± 152 *	377 ± 29 *	139 ± 13 *
	GCWB1085-low	994 ± 148 *	461 ± 74 *	919 ± 51 *	13.4 ± 0.3 **	2693 ± 360 *	581 ± 48 *	97 ± 7.9 *
	GCWB1085-high	800 ± 46 *	150 ± 23 *	829 ± 43 *	12.7 ± 0.2 *	2193 ± 221 *	436 ± 41 *	115 ± 8.5 *
	GCWB1156-low	915 ± 56 *	475 ± 120 *	892 ± 36 *	13.0 ± 0.3 *	2600 ± 431 *	504 ± 36 *	95 ± 5.2 *
	GCWB1156-high	841 ± 51 *	155 ± 23 *	829 ± 37 *	12.6 ± 0.3 *	1922 ± 319 *	379 ± 23 *	120 ± 9.5 *
	Synatura (200 mg/kg)	714 ± 33 *	87 ± 11 *	784 ± 53 *	12.5 ± 0.2 *	1722 ± 266 *	336 ± 37 *	130 ± 11 *

BALF was collected 24 h after the last airway challenge. The levels of cytokines in BALF were measured by enzyme-linked immunosorbent assay. Synatura was used as a positive control. Data are expressed as means ± SD (n = 8). # *p* < 0.001 compared with the control group. ## *p* < 0.001 compared with the OVA group. * *p* < 0.001 compared with the OVA plus DEPM group. ** *p* < 0.01 compared with the OVA plus DEPM group. TNF: tumor necrosis factor; IL: interleukin; IFN: interferon; MCP: monocyte chemoattractant protein.

## Abbreviations

AHR	Airway hyperresponsiveness
B(a)P	Benzo(a)pyrene
BALF	Bronchoalveolar lavage fluid
DEPM	Diesel exhaust particulate matter
ELISA	Enzyme-linked immunosorbent assay
LAB	Lactic acid bacteria
OVA	Ovalbumin
PAHs	Polycyclic aromatic hydrocarbons
PM	Particulate matter

## References

[1] Boulet L.P. (2018). Airway remodeling in asthma: Update on mechanisms and therapeutic approaches. Curr. Opin. Pulm. Med..

[2] Olin J.T., Wechsler M.E. (2014). Asthma: Pathogenesis and novel drugs for treatment. BMJ.

[3] Kim H.Y., DeKruyff R.H., Umetsu D.T. (2010). The many paths to asthma: Phenotype shaped by innate and adaptive immunity. Nat. Immunol..

[4] Murphy D.M., O’Byrne P.M. (2010). Recent advances in the pathophysiology of asthma. Chest.

[5] Steiner S., Bisig C., Petri-Fink A., Rothen-Rutishauser B. (2016). Diesel exhaust: Current knowledge of adverse effects and underlying cellular mechanisms. Arch. Toxicol..

[6] Muñoz X., Barreiro E., Bustamante V., Lopez-Campos J.L., González-Barcala F.J., Cruz M.J. (2019). Diesel exhausts particles: Their role in increasing the incidence of asthma. Reviewing the evidence of a causal link. Sci. Total Environ..

[7] Ghio A.J., Smith C.B., Madden M.C. (2012). Diesel exhaust particles and airway inflammation. Curr. Opin. Pulm. Med..

[8] Guarnieri M., Balmes J.R. (2014). Outdoor air pollution and asthma. Lancet.

[9] Kechagia M., Basoulis D., Konstantopoulou S., Dimitriadi D., Gyftopoulou K., Skarmoutsou N., Fakiri E.M. (2013). Health benefits of probiotics: A review. ISRN Nutr..

[10] Kalliomaki M., Salminen S., Poussa T., Arvilommi H., Isolauri E. (2013). Probiotics and prevention of atopic disease: 4-year followup of a randomised placebo-controlled trial. Lancet.

[11] Wohlgemuth S., Loh G., Blaut M. (2010). Recent developments and perspectives in the investigation of probiotic effects. Int. J. Med. Microbiol..

[12] Popova M., Molimard P., Courau S., Crociani J., Dufour C., Le Vacon F., Carton T. (2012). Beneficial effects of probiotics in upper respiratory tract infections and their mechanical actions to antagonize pathogens. J. Appl. Microbiol..

[13] Żukiewicz-Sobczak W., Wróblewska P., Adamczuk P., Silny W. (2014). Probiotic lactic acid bacteria and their potential in the prevention and treatment of allergic diseases. Cent. Eur. J. Immunol..

[14] Jan R.L., Yeh K.C., Hsieh M.H., Lin Y.L., Kao H.F., Li P.H., Chang Y.S., Wang J.Y. (2012). *Lactobacillus gasseri* suppresses Th17 proinflammatory response and attenuates allergen-induced airway inflammation in a mouse model of allergic asthma. Br. J. Nutr..

[15] Julia V., Macia L., Dombrowicz D. (2015). The impact of diet on asthma and allergic diseases. Nat. Rev. Immunol..

[16] Nawaz M., Ma C., Basra M.A., Wang J., Xu J. (2015). Amelioration of ovalbumin induced allergic symptoms in Balb/c mice by potentially probiotic strains of lactobacilli. Benef. Microbes.

[17] Wu C.T., Lin F.H., Lee Y.T., Ku M.S., Lue K.H. (2019). Effect of *Lactobacillus rhamnosus* GG immunopathologic changes in chronic mouse asthma model. J. Microbiol. Immunol. Infect..

[18] Li C.Y., Lin H.C., Hsueh K.C., Wu S.F., Fang S.H. (2010). Oral administration of *Lactobacillus salivarius* inhibits the allergic airway response in mice. Can. J. Microbiol..

[19] Wang X., Hui Y., Zhao L., Hao Y., Guo H., Ren F., Wang X. (2017). Oral administration of *Lactobacillus paracasei* L9 attenuates PM2.5-induced enhancement of airway hyperresponsiveness and allergic airway response in murine model of asthma. PLoS ONE.

[20] Pang L., Zou S., Shi Y., Mao Q., Chen Y. (2019). Apigenin attenuates PM_2.5_-induced airway hyperresponsiveness and inflammation by down-regulating NF-κB in murine model of asthma. Int. J. Clin. Exp. Pathol..

[21] Wei T., Tang M. (2018). Biological Effects of Airborne Fine Particulate Matter (PM_2.5_) Exposure on pulmonary immune system. Environ. Toxicol. Pharmacol..

[22] Mei M., Song H., Chen L., Hu B., Bai R., Xu D., Liu Y., Zhao Y., Chen C. (2018). Early-life exposure to three size-fractionated ultrafine and fine atmospheric particulates in Beijing exacerbates asthma development in mature mice. Part. Fibre Toxicol..

[23] Chen J.C., Tsai C.C., Hsieh C.C., Lan A., Huang C.C., Leu S.F. (2018). Multispecies probiotics combination prevents ovalbumin-induced airway hyperreactivity in mice. Allergol. Immunopathol. (Madr).

[24] Kim W.G., Kang G.D., Kim H.I., Han M.J., Kim D.H. (2019). *Bifidobacterium longum* IM55 and *Lactobacillus plantarum* IM76 alleviate allergic rhinitis in mice by restoring Th2/Treg imbalance and gut microbiota disturbance. Benef. Microbes.

[25] Li N., Xia T., Nel A.E. (2008). The role of oxidative stress in ambient particulate matter induced lung diseases and its implications in the toxicity of engineered nanoparticles. Free Radic. Biol. Med..

[26] Chauhan P.S., Dash D., Singh R. (2017). Intranasal Curcumin Inhibits Pulmonary Fibrosis by Modulating Matrix Metalloproteinase-9 (MMP-9) in Ovalbumin-Induced Chronic Asthma. Inflammation.

[27] Morales-Bárcenas R., Chirino Y.I., Sánchez-Pérez Y., Osornio-Vargas Á.R., Melendez-Zajgla J., Rosas I., García-Cuellar C.M. (2015). Particulate matter (PM_10_) induces metalloprotease activity and invasion in airway epithelial cells. Toxicol. Lett..

[28] Gauvreau G.M., Ellis A.K., Denburg J.A. (2009). Haemopoietic processes in allergic disease, eosinophil/basophil development. Clin. Exp. Allergy.

[29] Uhm T.G., Kim B.S., Chung I.Y. (2012). Eosinophil development, regulation of eosinophil-specific genes, and role of eosinophils in the pathogenesis of asthma. Allergy Asthma Immunol. Res..

[30] Wang P., Nie X., Wang Y., Li Y., Ge C., Zhang L., Wang L., Bai R., Chen A., Zhao Y. (2013). Multiwall carbon nanotubes mediate macrophage activation and promote pulmonary fibrosis through TGF-beta/Smad signaling pathway. Small.

[31] Ozdemir O. (2010). Various effects of different probiotic strains in allergic disorders: An update from laboratory and clinical data. Clin. Exp. Immunol..

[32] Liu Y.W., Liao T.W., Chen Y.H., Chiang Y.C., Tsai Y.C. (2014). Oral administration of heat-inactivated Lactobacillus plantarum K37 modulated airway hyperresponsiveness in ovalbumin-sensitized BALB/c mice. PLoS ONE.

[33] Di Gangi A., Di Cicco M.E., Comberiati P., Peroni D.G. (2020). Go With Your Gut: The Shaping of T-Cell Response by Gut Microbiota in Allergic Asthma. Front. Immunol..

[34] Li L., Fang Z., Liu X., Hu W., Lu W., Lee Y.K., Zhao J., Zhang H., Chen W. (2020). Lactobacillus reuteri attenuated allergic inflammation induced by HDM in the mouse and modulated gut microbes. PLoS ONE.

[35] Comhair S.A., Xu W., Ghosh S., Thunnissen F.B., Almasan A., Calhoun W.J., Janocha A.J., Zheng L., Hazen S.L., Erzurum S.C. (2005). Superoxide dismutase inactivation in pathophysiology of asthmatic airway remodeling and reactivity. Am. J. Pathol..

[36] Imaoka H., Hoshino T., Okamoto M., Sakazaki Y., Sawada M., Takei S., Kinoshita T., Kawayama T., Kato S., Aizawa H. (2009). Endogenous and exogenous thioredoxin 1 prevents goblet cell hyperplasia in a chronic antigen exposure asthma model. Allergol. Int..

[37] Voynow J.A., Fischer B.M., Malarkey D.E., Burch L.H., Wong T., Longphre M., Ho S.B., Foster W.M. (2004). Neutrophil elastase induces mucus cell metaplasia in mouse lung. Am. J. Physiol. Lung Cell. Mol. Physiol..

[38] De Marco V.G., Habibi J., Whaley-Connell A.T., Schneider R.I., Sowers J.R., Andresen B.T., Gutweiler A.A., Ma L., Johnson M.S., Ferrario C.M. (2009). Rosuvastatin ameliorates the development of pulmonary arterial hypertension in the transgenic (mRen2)27 rat. Am. J. Physiol. Heart Circ. Physiol..

[39] Li N., Wang M., Bramble L.A., Schmitz D.A., Schauer J.J., Sioutas C., Harkema R.H., Nel A.E. (2009). The adjuvant effect of ambient particulate matter is closely reflected by the particulate oxidant potential. Environ. Health Perspect..

[40] Huang K.L., Liu S.Y., Chou C.C., Lee Y.H., Cheng T.J. (2017). The effect of size segregated ambient particulate matter on Th1/Th2-like immune responses in mice. PLoS ONE.

[41] Lee E.G., Rhee C.K. (2020). The clinical efficacy of AG NPP709 (Synatura^®^) in patients with chronic bronchitis type stable chronic obstructive pulmonary disease. J. Thorac. Dis..

[42] Li N., Russell W.M., Douglas-Escobar M., Hauser N., Lopez M., Neu J. (2009). Live and heat-killed *Lactobacillus rhamnosus* GG: Effects on proinflammatory and anti-inflammatory cytokines/chemokines in gastrostomy-fed infant rats. Pediatr. Res..

[43] Choi S.S., Kim Y., Han K.S., You S., Oh S., Kim S.H. (2006). Effects of *Lactobacillus* strains on cancer cell proliferation and oxidative stress in vitro. Lett. Appl. Microbiol..

[44] Kobatake E., Nakagawa H., Seki T., Miyazaki T. (2017). Protective effects and functional mechanisms of *Lactobacillus gasseri* SBT2055 against oxidative stress. PLoS ONE.

[45] Gao D., Gao Z., Zhu G. (2013). Antioxidant effects of *Lactobacillus plantarum* via activation of transcription factor Nrf2. Food Funct..

[46] Saunders V., Breysse P., Clark J., Sproles A., Davila M., Wills-Karp M. (2010). Particulate matter-induced airway hyperresponsiveness is lymphocyte dependent. Environ. Health Perspect..

[47] Al-Ramli W., Prefontaine D., Chouiali F., Martin J.G., Olivenstein R., Lemiere C., Hamid Q. (2009). T(H)17-associated cytokines (IL-17A and IL-17F) in severe asthma. J. Allergy Clin. Immunol..

[48] Lee C.D., Lee J.K., Jeong J.H., Park E.S., Sohn U.D., Lee J.H., Shin J.W., Lee J.Y. (2018). Inhibitory effects of an extract mixture of ivy (*Hedera helix*) leaves and *Coptidis rhizoma* on ovalbumin-induced allergic lung inflammation by co-exposure to Asian sand dust in mice. Yakhak Hoeji.

[49] Yao X.J., Huang K.W., Li Y., Zhang Q., Wang J.J., Wang W., Liu J., Lv Z., An Y.Q., Ding Y.Z. (2014). Direct comparison of the dynamics of IL-25- and ’allergen ’-induced airways inflammation, remodelling and hypersensitivity in a murine asthma model. Clin. Exp. Allergy.

[50] Ip W.K., Wong C.K., Lam C.W. (2006). Interleukin (IL)-4 and IL-13 up-regulate monocyte chemoattractant protein-1 expression in human bronchial epithelial cells: Involvement of p38 mitogen-activated protein kinase, extracellular signal-regulated kinase 1/2 and Janus kinase-2 but not c-Jun NH2-terminal kinase 1/2 signalling pathways. Clin. Exp. Immunol..

